# Localization and Functional Characterization of the Rat Oatp4c1 Transporter in an *In Vitro* Cell System and Rat Tissues

**DOI:** 10.1371/journal.pone.0039641

**Published:** 2012-06-29

**Authors:** Kuei-Ling Kuo, Haining Zhu, Patrick J. McNamara, Markos Leggas

**Affiliations:** 1 Department of Pharmaceutical Sciences, College of Pharmacy, University of Kentucky, Lexington, Kentucky; 2 Department Molecular and Cellular Biochemistry, College of Medicine, University of Kentucky, Lexington, Kentucky; University of Pittsburgh, United States of America

## Abstract

The organic anion transporting polypeptide 4c1 (Oatp4c1) was previously identified as a novel uptake transporter predominantly expressed at the basolateral membrane in the rat kidney proximal tubules. Its functional role was suggested to be a vectorial transport partner of an apically-expressed efflux transporter for the efficient translocation of physiological substrates into urine, some of which were suggested to be uremic toxins. However, our *in vitro* studies with MDCKII cells showed that upon transfection rat Oatp4c1 polarizes to the apical membrane. In this report, we validated the trafficking and function of Oatp4c1 in polarized cell systems as well as its subcellular localization in rat kidney. Using several complementary biochemical, molecular and proteomic methods as well as antibodies amenable to immunohistochemistry, immunofluorescence, and immunobloting we investigated the expression pattern of Oatp4c1 in polarized cell systems and in the rat kidney. Collectively, these data demonstrate that rat Oatp4c1 traffics to the apical cell surface of polarized epithelium and localizes primarily in the proximal straight tubules, the S3 fraction of the nephron. Drug uptake studies in Oatp4c1-overexpressing cells demonstrated that Oatp4c1-mediated estrone-3-sulfate (E3S) uptake was pH-dependent and ATP-independent. These data definitively demonstrate the subcellular localization and histological location of Oatp4c1 and provide additional functional evidence that reconciles expression-function reports found in the literature.

## Introduction

The kidney is responsible for homeostasis of endogenous and exogenous substances through tubular secretion and reabsorption, which in part is mediated by various membrane transporters, including the solute carrier family (SLC) and ATP-binding cassette (ABC) superfamily. Several studies have demonstrated that overlapping substrate specificity among uptake and efflux transporters is likely to accelerate the translocation of endogenous and exogenous substances across epithelial or endothelial barriers [1], [2]. Three members of the rodent *Oatp* family have been identified in the rat kidney proximal tubules. Oatp1a1 (previously: Oatp1) and Oatp1a3 (previously: Oat-k1, Oat-k2) are expressed at apical membranes [3], [4], and Oatp4c1 (previously: Oatp-H) was reported to be expressed at the basolateral membrane of the proximal tubule [5]. OATP4C1 is the only OATP detected in renal proximal tubules [6]. The reported substrates of OATP4C1 include cardiac glycosides (digoxin and ouabain), thyroid hormones (triiodothyronine (T_3_) and thyroxine (T_4_)), cAMP, methotrexate (MTX) [5], sitagliptin [7], and estrone-3-sulfate (E3S) [8]. The transporter has been shown to have unidirectional uptake function, but the driving force has not yet been elucidated. Mikkaichi et al. showed that sodium, chloride ion, and pH do not affect OATP4C1-mediated uptake, while ATP depletion partially inhibits T_3_ uptake to 40% [5]. In contrast, Leuthold et al. demonstrated that OATP4C1-mediated E3S or T_4_ uptake is significantly higher at extracellular pH 6.5 than pH 8.0 [9]. In addition, OATP4C1 has been suggested to possess multiple substrate recognition sites, because digoxin does not inhibit OATP4C1-mediated T_3_ [5] or E3S [8] uptake and vice versa, while E3S and T_3_ have mutual inhibition. Furthermore, the physiological role of OATP4C1, reportedly a basolateral uptake transporter, has been postulated to be coupling with P-glycoprotein, an apical efflux transporter, to facilitate the renal clearance of common substrates such as digoxin [5] and uremic toxins [10]. In studies with transgenic rats harboring human SLCO4C1, the decrease of uremic toxin (guanidino succinate, asymmetric dimethylarginine, and trans-aconitate) concentrations in plasma suggests that OATP4C1 may facilitate the excretion of uremic toxins in renal failure models and, by extension, in patients with chronic kidney disease. However, direct evidence that these toxins are OATP4C1 substrates is lacking.

In addition to the kidney, there are limited data regarding the expression of OATP4C1. Microarray expression has shown that human SLCO4C1 is also expressed in the liver, lung, mammary gland, skin, neutrophils, peripheral leukocytes and mononuclear cells [6]. Rat Slco4c1 is detected mainly in the kidney and lung, and slightly in the brain by Northern Blot analysis [5]. To further study the role of this transporter in drug disposition, we sought to generate transfected cell lines expressing rat Oatp4c1. Initial attempts in our laboratory to generate Oatp4c1-expressing MDCKII cells produced unexpected results as the transporter localized at the apical rather than the basolateral membranes. Although this result was unexpected, it is not without precedent. Previous work by Lai and Tan showed that MRP4 was localized in the basolateral membrane of MDCKII cells [11]. However, MRP4 localizes at the apical membrane of human and rodent renal tubules [12]. This discrepancy was later attributed to species differences in Na^+^/H^+^ exchanger regulatory factor 1 (NHERF1) expression, an adaptor protein, which determines the trafficking of MRP4 [13]. In this study we sought to definitively demonstrate the subcellular localization of Oatp4c1 in the *in vitro* models and in rat tissues. We used a comprehensive approach including multiple antibodies amenable to immunohistochemistry, immunofluorescence, and immunobloting to probe the expression of rat Oatp4c1 in intact tissues and cells as well as biochemically separated and enriched apical and basolateral membranes isolated from polarized cells and renal proximal tubules. Furthermore, proteomic analysis was used to qualitatively validate the specificity of our antibody. Functional activity of Oatp4c1 in MDCKII-Oatp4c1 was probed with E3S. Collectively, our data provide evidence that is contrary to published work regarding the localization, polarity, and function of Oatp4c1.

## Methods

### Ethics Statement

Tissues from animals were collected using procedures formally approved by the University of Kentucky Institutional Animal Care and Use Committee protocol# 2007-0228. All cell culture procedures and cell-line modifications were performed with approval from the Institutional Biosafety committee at the University of Kentucky under application B07-598.

### Chemicals

[^3^H]-E3S (45.6 Ci/mmol) was from Perkin Elmer (Waltham, MA). GF120918 was a gift from GlaxoSmithKline (Research Triangle Park, NC). All other chemicals were purchased from Sigma-Aldrich (St. Louis, MO) unless specified otherwise. All procedures involving the culture and manipulations of cell lines were approved by the University of Kentucky Biological Safety Committee.

### Antibodies

Rabbit polyclonal anti-Oatp4c1 antibodies were generated (Open Biosystems, Rockford, IL) against sequence peptides of rat Oatp4c1. Two amino-terminus [KGVENPAFVPSSPDTPRR (PA1254); QPRESEDPQKSTEPS (F3608)], two carboxyl-terminus [STITVEEDLNKIENEG (PA1343); TVEEDLNKIENEG (PA1562)], and an extracellular loop [SPDFEARAGKC (PA1556)] previously reported by Mikkaichi and colleagues [5]. PA1343 was affinity-purified. Rabbit polyclonal anti-Abcg2 antibodies were generated (Open Biosystems) against Walker A peptide (GKSSLLDVLAARKD) of rat Abcg2 [14] and were used to determine the expression of Abcg2 in brush border membranes by western blot. Rat monoclonal antibody against E-cadherin (DECMA-1), mouse monoclonal antibodies against calbindin-D_28k_ (CB-955) and aquaporin 1 (1/22) were obtained from Abcam (Cambridge, MA). Mouse monoclonal antibody against ZO-1 (zo1-1A12) was from Invitrogen. Mouse monoclonal antibody against β-actin (AC-15) was from Sigma-Aldrich. Mouse monoclonal antibody against P-gp (C219) was from Signet (Dedham, MA). Rat monoclonal antibody against rat Abcg2 (BXP-53) and Mrp4 (M_4_I-10) were from Kamiya (Seattle, WA). Rabbit polyclonal antibody against Na^+^/K^+^-ATPase (#3010) was obtained from Cell Signaling (Danvers, MA).

### Construction of Plasmid Vector

Total RNA was isolated from a frozen kidney of a Sprague-Dawley rat using RNeasy Kit (Qiagen, Valencia, CA) as directed by the manufacture including on-column DNA digestion, and then reverse-transcribed to cDNA using ThermoScript™ RT-PCR kit (Invitrogen) with oligo (dT)_20_ primers according to the manufacturer’s protocol. Rat Slco4c1 cDNA was amplified using Phusion High-Fidelity PCR kit (New England Biolabs, Ipswich, MA) with forward (5′- CACCATGCAGGGTTCCAAGGGAG-3′) and reverse (5′- CTATCCTTCGTTCTCTATTTTGTTG-3′) primers. Amplification conditions were as follows: 98°C (1 min), [98°C (10 sec), 64°C (30 sec), 72°C (35 sec)] × 40 cycles, 72°C (10 min). The amplified fragment was purified using Zymoclean™ Gel DNA Recovery kit (Zymo Research, Irvine, CA). The subsequent sequence was cloned into the vector pENTR (Invitrogen) and the construct was transformed into competent One Shot TOP10 *E. coli* cells (Invitrogen). The plasmid DNA was isolated from a positive transformant using QIAprep Spin Miniprep Kit (Qiagen) according to the manufacturer’s protocol. The sequence was confirmed (Davis sequencing, Davis, CA) to share 100% identity with rat Slco4c1 sequence (GeneBank accession number NM_001002024). The plasmid DNA was then subcloned into the multiple cloning site of the mammalian expression vector pcDNA6.2/GFP-DEST (Invitrogen).

### Cell Culture and Transfection

Madin-Darby canine kidney II (MDCKII) cells (European Collection of Cell Cultures, Salisbury, UK) were cultured in minimum essential medium with Earle’s salts (MEM, Mediatech, Manassas, VA) supplemented with 5% fetal bovine serum (Atlanta Biologicals, Lawrenceville, GA), 100 U/ml penicillin, and 100 µg/ml streptomycin (Invitrogen) at 37°C in a humidified incubator with 5% CO_2_. LLC-PK1 cells (kind gift from Dr. Alferd Schinkel [15], Netherlands Cancer Institute) were cultured in Dulbecco’s modified Eagle’s medium (DMEM, Mediatech) supplemented with 10% fetal bovine serum, 100 U/ml penicillin, and 100 µg/ml streptomycin. The pcDNA6.2/GFP-DEST-rat Slco4c1 and pcDNA6.2/GFP-DEST were transfected into MDCKII and LLC-PK1 cells using FuGENE6 reagent (Roche Diagnostics Co., Indianapolis, IN) according to the manufacturer’s instructions. The transfected cells were selected with 5 µg/ml blasticidin (Invitrogen). It should be noted that under these experimental conditions pcDNA6.2/GFP-DEST expresses only the gene of interest (Slco4c1) because we did not use Tag-On-Demand Suppressor Supernatant (Invitrogen) to induce a GFP fused Oatp4c1. Rat Oatp4c1 expression was assessed by western blot analysis and immunohistochemical analysis. The subcellular localization in pooled cells was examined by surface protein biotinylation assay (described below), and confocal microscopy. A clone with the highest Oatp4c1 expression was obtained by serial dilution and was used for uptake studies.

### Western Blot Analysis

Cell lysates were prepared from Oatp4c1-expressing and vector-transfected MDCKII cells. Following a wash with ice-cold PBS, cells were scraped and pelleted by centrifugation at 300 g for 5 min. Cell pellets were resuspended in RIPA buffer (50 mM Tris, 150 mM NaCl, 1% Nonidet P-40, 0.5% deoxycholate, 1 mM EDTA, 0.1% SDS, pH 8.0) with protease inhibitor cocktail (Roche Diagnostics Co.) and shaken at 4°C for 30 min.

Crude membrane fractions were prepared from Sprague-Dawley female rat tissues. The tissues were disrupted in Dounce Buffer (10 mM Tris, 0.5 mM MgCl_2_, protease inhibitor cocktail, pH 7.6) with brief pulses of sonication using a probe ultrasonic processor (Thermo Fisher Scientific) at 4°C and tonicity was restored to 150 mM with NaCl. Following 300 g centrifugation for 5 min, EDTA was added to the supernatant to a final concentration of 5 mM. The samples were centrifuged at 100,000 g for 1 hr at 4°C. Pellets were resuspended in buffer (0.2 M mannitol, 0.07 M sucrose, 50 µM Tris HCl, 1 µM EDTA). Protein concentrations were measured using a bicinchoninic acid assay (BCA Protein Assay Kit, Thermo Fisher Scientific, Waltham, MA). Cell lysates and crude membrane fractions were stored at −80°C until use.

Cell lysates and crude membrane fractions were mixed with NuPAGE LDS sample buffer (Invitrogen) and 0.1 M dithiothreitol before incubation at 70°C for 10 min. Proteins were separated on a 10% SDS-polyacrylamide gel with 5% stacking gel and transferred to a polyvinylidene difluoride membrane (Millipore, Billerica, MA) at 200 mA for 2 hr at 4°C. Following blocking with 5% skimmed milk in phosphate buffered saline with 0.05% Tween-20 (PBST) for 3 hr at room temperature, the membranes were incubated with 55 µg/ml anti-Oatp4c1 polycolonal antibody (PA1343) in 5% skimmed milk in PBST overnight at 4°C. After washing with PBST, membranes were incubated with HRP conjugated goat anti-rabbit IgG (Thermo Fisher Scientific) in 5% skimmed milk in PBST for 1 hr at room temperature. Following washing with PBST, a standard ECL kit (Thermo Fisher Scientific, Rockford, IL) and Image Station 2000 MM (Eastman Kodak, Rochester, NY) were used for visualization.

### Immunofluorescence Microscopy

Oatp4c1-expressing and vector-transfected MDCKII cells were grown on Snapwell polycarbonate filter membranes (0.4 µm pore size, 1.12 cm^2^ growth area, Corning Costar, Lowell, MA) for 4 days to achieve transepithelial electrical resistance >200 Ω•cm^2^. Filter membranes were washed with ice-cold PBS, excised and processed for staining. After fixation with −20°C methanol for 10 min, rehydration with PBS for 5 min, permeabilization (0.2% triton X-100 in PBS) for 15 min, and blocking with 10% goat serum for 1 hr, cells were incubated with the rabbit anti-Oatp4c1 polyclonal antibody (PA1343, 110 µg/ml) and mouse anti-ZO-1 monoclonal antibody (5 µg/ml) for 2 hr at room temperature. Following washes with permeabilization solution, cells were incubated with 4 µg/ml Alexa Fluor 568 goat anti-rabbit IgG and Alexa Fluor 488 anti-mouse IgG (Invitrogen) for 1 hr at room temperature. Cells were washed with permeabilization solution and mounted on glass slides with Prolong Gold® containing DAPI solution (Invitrogen) to visualize nuclei. The fluorescence emission was visualized using Axiovert 200 M confocal microscope (Carl Zeiss, Thornwood, NY).

### Surface Protein Biotinylation Assay

Oatp4c1-expressing and vector-transfected MDCKII cells were grown on Transwell polycarbonate filter membranes (3 µm pore size, 4.67 cm^2^ growth area, Corning Costar) for 4 days to achieve transepithelial electrical resistance >200 Ω•cm^2^. Cells were washed with ice-cold PBS-Mg/Ca buffer (PBS buffer containing 0.1 mM CaCl_2_ and 1 mM MgCl_2_). Freshly prepared Sulfo-NHS-SS-Biotin (1 mg/ml in PBS with Mg^++^/Ca^++^) was added to either the apical or basolateral chamber and incubated with slow rocking at 4°C for 20 min. Cells were then washed and incubated with quenching buffer (5 mM glycine in PBS-Mg/Ca) with slow rocking at 4°C for 20 min to remove unreacted biotin. Following washing with PBS with Mg^++^/Ca^++^, cells were lysed with 700 µl lysis buffer (10 mM Tris, 150 mM NaCl, 1 mM EDTA, 0.1% SDS, 1% Triton X-100, pH 7.4, with protease inhibitor cocktail) for 30 min at 4°C with shaking. Cell lysates were centrifuged at 10,000 g for 2 min at 4°C and supernatants were incubated with streptavidin agarose beads with end-over-end rotation for 1 hr at 4°C. Samples were washed three times with lysis buffer and incubated with 50 µl 2X Laemmli Buffer (125 mM Tris, 10% glycerol, 2%SDS, 5% 2-β-mercaptoethanol, pH 6.8) for 30 min at room temperature. 2-β-mercaptoethanol was used to break the sulfhydryl (SS) bond between the biotin and the protein reactive group releasing the biotinylated membrane proteins from the beads. Samples were centrifuged at 10,000 g for 2 min, and the supernatants were subjected to western blot analysis.

### Immunohistochemistry

Female Sprague-Dawley rat liver and kidney were isolated and fixed in 4% paraformaldehyde for 6 hr at room temperature. The tissues were embedded in paraffin using standard histology techniques, and cut as 6 µm-thick sections. After deparaffinization and rehydration in xylene and ethanol gradient solutions, the antigen was retrieved under pressure in 10 mM citrate buffer, pH 6.0. Sections were blocked with 3% H_2_O_2_ in PBS for 10 min and treated with avidin and biotin blocking reagent (Vector Laboratories, Burlingame, CA) for 15 min each. Non-specific interactions were blocked with 2% normal goat serum in PBST for 30 min prior to incubating with 220 µg/ml anti-Oatp4c1 antibody (PA1343) for 24 hr at room temperature. For visualization, sections were incubated with 5 µg/ml biotinylated goat anti-rabbit IgG (Vector Laboratories) for 30 min at room temperature, Streptavidin-HRP reagent (Dako, Carpinteria, CA) for 20 min and NovaRED chromogen substrate (Vector Laboratories) for 5 min. After counterstaining with hematoxylin and dehydration, the sections were mounted with Vecta-Mount (Vector Laboratories).

To further study the localization of Oatp4c1, the rat tissue sections were double stained with anti-Oatp4c1 antibody (PA1343, 110 µg/ml) and the following antibodies: anti-E-cadherin (2 µg/ml), anti-AQP1 (10 µg/ml), and anti-calbindin-D_28k_ (40 µg/ml), anti-Abcg2 (BXP-53, 12.5 µg/ml), anti-Mrp4 (3 µg/ml), anti-P-gp (2.5 µg/ml). For visualization, the sections were then incubated with fluorescence conjugated secondary antibodies for 30 min at room temperature and then mounted with Prolong Gold® DAPI containing solution. The fluorescence emission was visualized using Axiovert 200 M confocal microscope (Carl Zeiss, Thornwood, NY).

### BBM (Brush-border Membrane) and BLM (Basolateral Membrane) Isolation

The BBM and BLM were isolated by Percoll density gradient centrifugation according to procedures described by Goldinger et al. with some modifications [16]. Briefly, two male Sprague-Dawley rats were euthanized by rapid decapitation and the kidneys were excised and frozen immediately in liquid nitrogen. Frozen kidney cortex tissues were excised and homogenized in sucrose buffer (250 mM sucrose, 10 mM triethanolamine, pH 7.4, protease inhibitor cocktail) with a motorized homogenizer. Samples were centrifuged at 2,500 g for 15 min at 4°C. The supernatant was centrifuged at 20,000 g for 20 min at 4°C. The less dense top layer of the pellet was collected and the lower dark segment of the pellet was discarded. The pellet was resuspended in 26.5 ml sucrose buffer with 10 strokes of a tight plunger in a Dounce homogenizer. Following addition of 3.5 ml Percoll the suspension was mixed by inversion and centrifuged at 48,000 g for 30 min at 4°C. Two distinct bands were formed and carefully isolated. The upper band was BLM and the lower one was BBM [16]. An equal volume of sucrose buffer was added to each fraction, and the Percoll was removed by centrifuging at 93000 g for 1 hr at 4°C. The single band of membrane proteins was collected and the glassy Percoll pellet was discarded. The BLM was resuspended in mannitol buffer (150 mM mannitol, 2.5 mM EGTA, 6 mM HEPES, pH 7.4). The BBM pellet was further purified by magnesium precipitation [17]. Following resuspension in buffer (100 mM mannitol, 1.6 mM EGTA, 0.3 mM PMSF, 0.3 mM sodium orthovanadate, 4 mM HEPES, pH 7.4), MgCl_2_ was added to yield a final concentration of 12 mM. The mixture was incubated on ice for 15 min with intermittent and gentle mixing. Remaining cellular debris were removed by centrifuging at 3,000 g for 10 min at 4°C, the resultant supernatant was centrifuged at 30,000 g for 40 min at 4°C to isolate membrane proteins. The BBM pellet was resuspended in mannitol buffer and again precipitated by addition of 12 mM MgCl_2_ as described above. The final pellet was resuspended in mannitol buffer. The BBM and BLM were stored at −80°C until use. Separation efficiency was evaluated by western blot analysis with anti- Na^+^/K^+^-ATPase (1∶1000), anti-Mrp4 (3 µg/ml) and anti-Abcg2 (150 µg/m).

### Protein Trypsin Digestion and LC-MS/MS Analysis

Approximately 100 µg of proteins from the BBM and BLM were subjected to 10% SDS-PAGE separation. The protein gel bands at ∼65–100 and ∼100–150 kDa were excised and underwent dithiothreitol reduction, iodoacetamide alkylation, and in-gel trypsin digestion using a standard protocol as previously reported [18], [19]. The resulting tryptic peptides were extracted, concentrated to 15 µl each using a SpeedVac, and 5 µl was injected for nano-LC-MS/MS analysis. LC-MS/MS data were acquired on an LTQ Velos Orbitrap mass spectrometer (Thermo Fisher Scientific, Waltham, MA) coupled with a Nano-LC Ultra/cHiPLC-nanoflex HPLC system (Eksigent, Dublin, CA) through a nano-electrospray ionization source. The tryptic peptides sample was injected by an autosampler, desalted on a trap column, and subsequently separated by reverse phase C18 column (75 µm i.d.×150 mm) at a flow rate of 250 nL/min. The HPLC gradient was linear from 5% to 60% mobile phase B for 30 min using mobile phase A (H_2_O, 0.1% formic acid) and mobile B (90% acetonitrile, 0.1% formic acid). Eluted peptides were analyzed using data-dependent acquisition: peptide mass spectrometry was obtained by Orbitrap with a resolution of 60,000. The seven most abundant peptides were subjected to collision induced dissociation and MS/MS analysis in LTQ linear trap. The LC-MS/MS data were submitted to a local MASCOT server for MS/MS protein identification search via the ProteomeDiscoverer software. The mass error tolerance was 5 ppm for peptide MS and 0.8 Da for MS/MS. All peptides were required to have an ion score greater than 30 (P<0.05). The false discovery rate in each LC-MS/MS analysis was set to be less than 1%.

### [^3^H]-E3S Uptake and Inhibition Studies

Oatp4c1-expressing and vector-transfected MDCKII cells were grown in 6-well plates. The cell culture medium was replaced with that containing 5 mM sodium butyrate to induce transporter expression 24 hr prior the uptake study [20]. The uptake buffer used in all transport experiments contained 118 mM NaCl, 23.8 mM NaHCO_3_, 4.83 mM KCl, 0.96 mM KH_2_PO_4_, 1.20 mM MgSO_4_, 5.0 mM glucose and 1.53 mM CaCl_2_. Buffer pH was adjusted to 7.4 by supplementation with 12.5 mM HEPES and pH adjustment with 1 M Tris-base. Alternatively, the buffer was supplemented with 12.5 mM 2-(N-morpholino)ethanesulfonic acid and pH was adjusted to 4.5 or 5.5 with 1 M Tris-base. Before the study, cells were washed once and preincubated with uptake buffer for 15 min at 37°C. Uptake was initiated by replacing the uptake buffer with that containing [^3^H]-E3S. After incubation for designated time at 37°C, cells were washed three times with ice-cold PBS, lysed with 0.5 ml of 0.5 N NaOH and neutralized with 0.1 ml of 2.5 N HCl. The intracellular radioactivity was determined by transferring 0.5 ml of lysate into scintillation cocktail (Research product international, Mt. Prospect, IL) for counting with a Packard 2200 CA Tri-Carb (PerkinElmer, Waltham, MA) liquid scintillation counter. Protein concentrations were measured using the BCA Protein Assay kit. Net uptake was calculated as the difference between [^3^H]-E3S uptake into Oatp4c1-expressing cells and uptake into the vector-transfected cells. For [^3^H]-E3S uptake under ATP-depletion conditions, cells were pre-incubated for 20 min prior to the experiment and during the experiment (1 min) with uptake buffer containing 20 mM 2-deoxy-D-glucose and 10 mM NaN_3_ without D-glucose [21]. For [^3^H]-E3S uptake inhibition, cells were pre-incubated for 15 min prior to and during the experiment (1 min) with 100 µM of various compounds at pH 5.5 or 7.4. Based on model fitting criteria, transport parameters were estimated using a sigmoid-E_max_ equation:
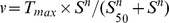
Where, ν, S, T_max_, S_50_, and *n* represent the initial uptake velocity, substrate concentration, maximum uptake transport velocity, Michaelis-Menten constant equivalent, and Hill coefficient, respectively. The model was fitted to the data (obtained in triplicate) by nonlinear regression using GraphPad Prism (GraphPad Software, San Diego, CA).

### Statistical Analysis

Values are presented as means ± S.D. Statistical analysis was performed with unpaired student’s t-test or two-way ANOVA and Bonferroni post-hoc test for multiple comparisons. p<0.05 was the criterion of statistical significance. GraphPad Prism was used for all statistical analysis (GraphPad Software, San Diego, CA).

## Results

### Expression and Polarity of Rat Oatp4c1 in Polarized Cell Lines

**Figure 1:**
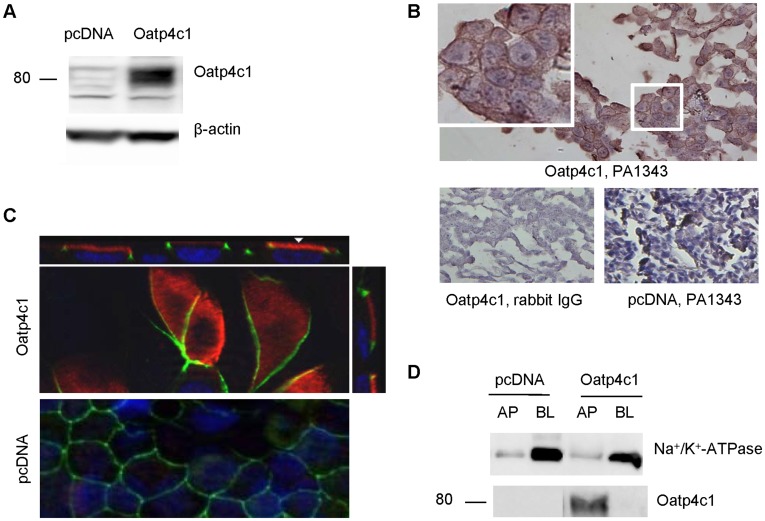
Validation of Oatp4c1 expression and subcellular localization in MDCKII cells. (A) Western blot analysis for Oatp4c1 expression. Protein lysates (50 µg per lane) from MDCKII cells transfected with pcDNA (empty vector) and Oatp4c1 were prepared from cells grown in monolayers. (B) Immunohistochemical analysis for Oatp4c1 expression. Paraformaldehyde-fixed paraffin-embedded cell sections were stained with PA1343. Color development with NovaRed signifies Oatp4c1 staining. All sections were counterstained with hemotoxylin. Rabbit IgG was used as a negative control. (C) Immunolocalization of Oatp4c1 in polarized MDCKII cells. Cells were double stained with Oatp4c1 (red) and ZO-1 (green). Nuclei were stained with DAPI (blue). Center image in the Oatp4c1 panel is a single optical section of the x–y plane while top and right images represent x–z and y–z planes, respectively, reconstructed from image stacks. The apical and basal sides can be demarcated by ZO-1 and the nuclei, respectively, in both x–z and y–z sections. (D) Western blot analysis of Oatp4c1 in proteins isolated from either the apical (AP) or basolateral (BL) membranes of polarized MDCKII cells. Surface proteins were isolated by biotin-streptavidin labeling on either the apical AP or BL compartment. Na^+^/K^+^-ATPase served as a BL marker to demonstrate the relative efficiency of AP and BL membrane separation. (Magnification (40×)).

To study the role of rat Oatp4c1 in drug disposition, we generated rat Oatp4c1-expressing MDCKII cells. The expression of Oatp4c1 in the transfected cells were verified by western blotting using the antibody (PA1343) generated against C-terminal of rat Oatp4c1 (Fig. 1A). Oatp4c1 expression was detectable as a band at ∼80 kDa in MDCKII-Oatp4c1 cells, but not in MDCKII-pcDNA (empty vector) cells. Expression was also confirmed in paraformaldehyde-fixed paraffin-embedded cell pellet sections by immunohistochemical analysis (Fig. 1B). Oatp4c1 was observed on the cell membrane (Fig. 1B, inset) in the MDCKII-Oatp4c1 cells, and no staining was observed in the negative control (rabbit IgG) or in the MDCKII-pcDNA cells. This demonstrated that the transfection was successful and that the antibody was specific.

Subsequently, we sought to determine the subcellular localization of Oatp4c1 in polarized cell monolayers by confocal immunofluorescence laser scanning microscopy (Fig. 1C). A tight-junction protein, ZO-1, was used as an apical marker. As shown in Fig. 1C, Oatp4c1 localizes at the apical membrane, along with ZO-1, in MDCKII-Oatp4c1 cells. This result was unexpected because Oatp4c1 was shown at the basolateral membrane in the rat proximal tubule [5]. This result was also confirmed by an alternate method. We used surface protein biotinylation to determine Oatp4c1 expression in membrane fractions obtained from polarized cell monolayers. The surface proteins on either apical or basolateral membranes were labeled with biotin and isolated from streptavidin agarose beads. Western blot analysis of the basolateral marker Na^+^/K^+^-ATPase demonstrated the efficient separation of the apical and basolateral membranes. Western blot analysis of Oatp4c1 demonstrated that the transporter was enriched in the apical membrane fraction, but not in the basolateral membrane (Fig. 1D). Collectively, these data verified the apical expression of the transporter in Oatp4c1-expressing MDCKII cells. However, there is precedent showing that a transporter can mis-localize in MDCKII cells. Expression studies in MRP4-transfected MDCKII cells show that MRP4 traffics to the basolateral membrane due to low expression of an adaptor protein, NHERF1, in these cells [11], [13]. However, MRP4 polarized correctly in NHERF1 expressing LLC-PK1 cells. While the adaptor proteins facilitating Oatp4c1 trafficking are not known, we transfected LLC-PK1 with pcDNA6.2-Oatp4c1 and determined its polarity. As observed in MDCKII cells, Oatp4c1 also polarized to the apical membrane in LLC-PK1 cells (Fig. S1).

**Figure 2:**
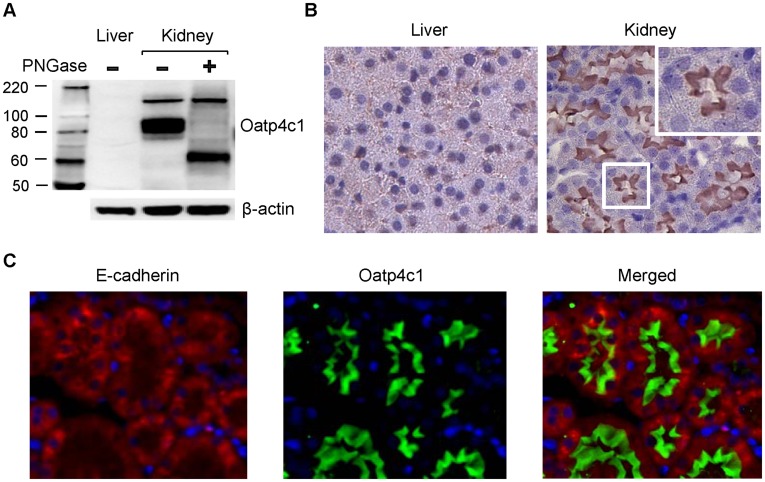
Oatp4c1 expression and localization in rat kidney. (A) Western blot analysis for Oatp4c1 expression in rat tissues. Crude membrane fractions from rat liver and kidney were prepared. PNGaseF was used for deglycosylation. Oatp4c1 was detected by PA1343. β-actin was used as a loading control. (B) Immunohistochemical analysis for Oatp4c1 subcellular localization in rat tissues. Paraformaldehyde-fixed paraffin-embedded rat tissue sections were stained with PA1343. Color development with NovaRed signifies Oatp4c1 staining. (C) Double immunofluorescence staining for Oatp4c1 (green) and E-cadherin (red) in rat kidney. E-cadherin served as a basolateral marker (Magnification: 40×).

Despite multiple efforts and approaches to generate a single clone of Oatp4c1 expressing MDCKII cells, immunofluorescence studies showed that expression was not detectable in at least 70% of the cells (Fig. S2A). Thus, to increase expression we treated cells with sodium butyrate, which is known to enhance protein expression. As shown in Fig. S2B and S2C, Slco4c1 mRNA and Oatp4c1 protein expression increased in the presence of 5 mM sodium butyrate, respectively. However, as shown in Fig. S2A the number of cells with Oatp4c1 expression was not significantly increased with sodium butyrate. To ensure that sodium butyrate induced expression did not interfere with the trafficking of Oatp4c1 we also performed immunofluorescence and demonstrated that the apical polarity was maintained (Fig. S2D).

### Expression and Subcellular Localization of Rat Oatp4c1 in the Tissues

Rat Oatp4c1 has been detected in the kidney, but not liver by northern blot analysis [5] or RT-PCR (data not shown). This result was confirmed by western blotting (Fig. 2A). A band was detected at ∼80 kDa and reduced to ∼65 kDa after deglycosylation, which is consistent with membrane protein post-translational modification. The band at ∼150 kDa did not shift after deglycosylation, indicating that it was not a specific band. In accord with the mRNA expression, no protein band was detected in the rat liver. The subcellular localization of Oatp4c1 was then assessed by immunohistochemical analysis in paraformaldehyde-fixed paraffin-embedded liver and kidney tissue sections. To assure the quality of the tissue specimens, the rat liver and kidney sections were stained with anti-Abcg2, and anti-Mrp4 antibody, respectively. Both tissues showed specific staining (data not shown). Consistent with the western blot result (Fig. 2A), Oatp4c1 was not detected in the rat liver, but Oatp4c1 stained strongly in apical membranes of kidney tubules (Fig. 2B). No staining was observed in tissue sections exposed to rabbit IgG, instead of primary antibody, and membrane Oatp4c1 staining was abolished when probing with antibody that was pre-absorbed with antigen peptide (Fig. S3). The distinct apical expression of Oatp4c1 in rat kidney was also demonstrated using dual staining for the basolateral marker E-cadherin [22] and Oatp4c1 (Fig. 2C).

**Figure 3:**
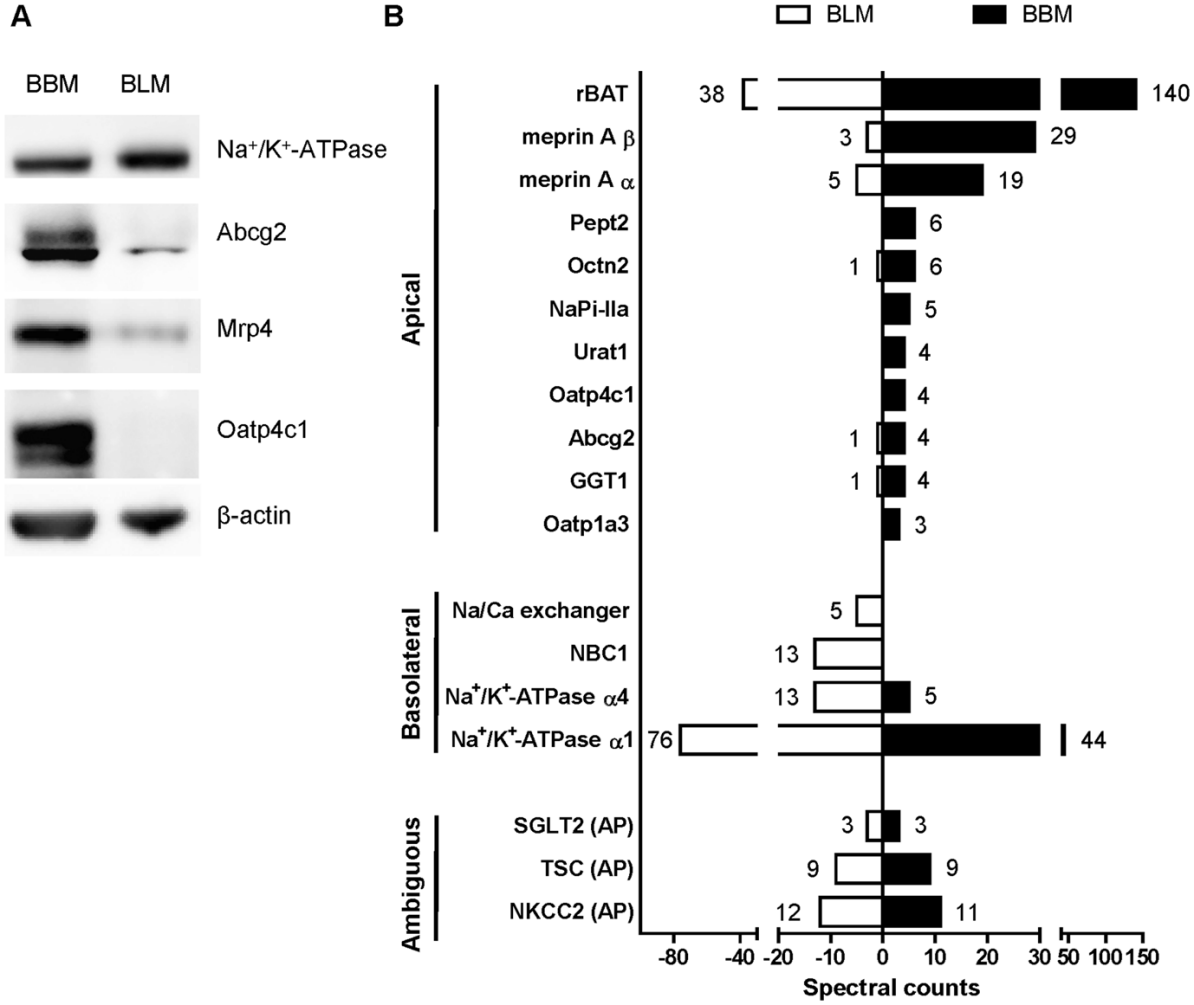
Expression of Oatp4c1 in isolated kidney brush border membrane (BBM) and basolateral membrane (BLM) fractions. (A) Western blot analysis for Oatp4c1 expression in BBM and BLM. Biochemical separation of membrane proteins (20 µg per lane) from rat kidney cortex was achieved by Percoll gradient. Na^+^/K^+^-ATPase alpha 1 was used as a basolateral marker. Abcg2 and Mrp4 served as apical markers. Oatp4c1 was detected by PA1343. (B) LC-MS/MS based proteomic analysis of ∼60–150 kD proteins isolated from BBM and BLM fractions. Spectral counts detected in protein digests obtained from ∼60–150 kD sections excised from the SDS-PAGE gel. Proteins with 3 or more spectral counts in one fraction are presented as “Apical” or “Basolateral” based on previously published data. Spectral counts indicate the number of unique peptides detected in each fraction. (AP, apical).

Given that these results were in contrast to previously reported expression pattern in the rat kidney, we sought to develop additional antibodies as well as the polyclonal antibody reported previously by Mikkaichi and colleagues [5]. As shown in Fig. S3, the apical Oatp4c1 expression was confirmed using three different antibodies (PA1562, PA1254, and F3608), but we were not able to observe staining, above background levels, with the antibody we developed using the peptide reported by Mikkaichi and colleagues [5].

**Figure 4:**
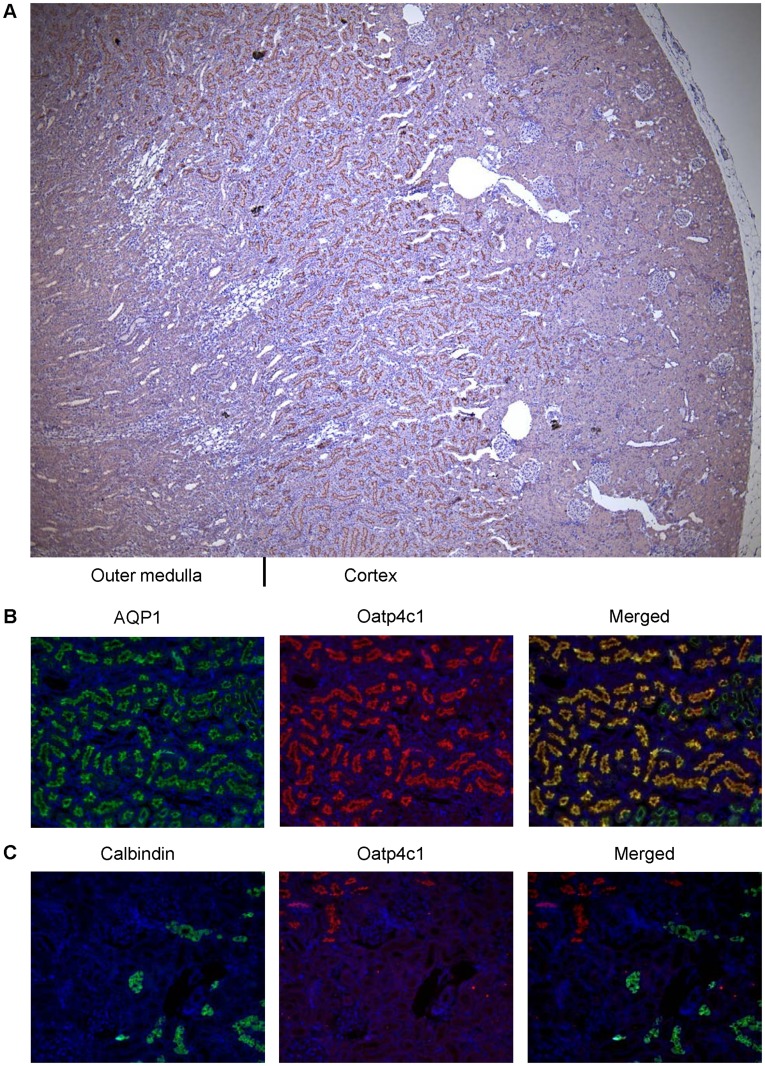
Oatp4c1 localization in paraformaldehyde-fixed paraffin-embedded rat kidney tissue sections. Paraformaldehyde-fixed paraffin-embedded kidney sections were stained with antibodies as indicated. Following staining with PA1343 antibody, color development with NovaRed signifies Oatp4c1 staining (A). Sections were double-stained with PA1343 (B, C, red) and the proximal tubule marker AQP1 (B, green) or the distal tubule marker calbindin (C, green). Nuclei (blue) were stained with DAPI (Magnification: 4× (A), 10× (B, C)).

**Figure 5:**
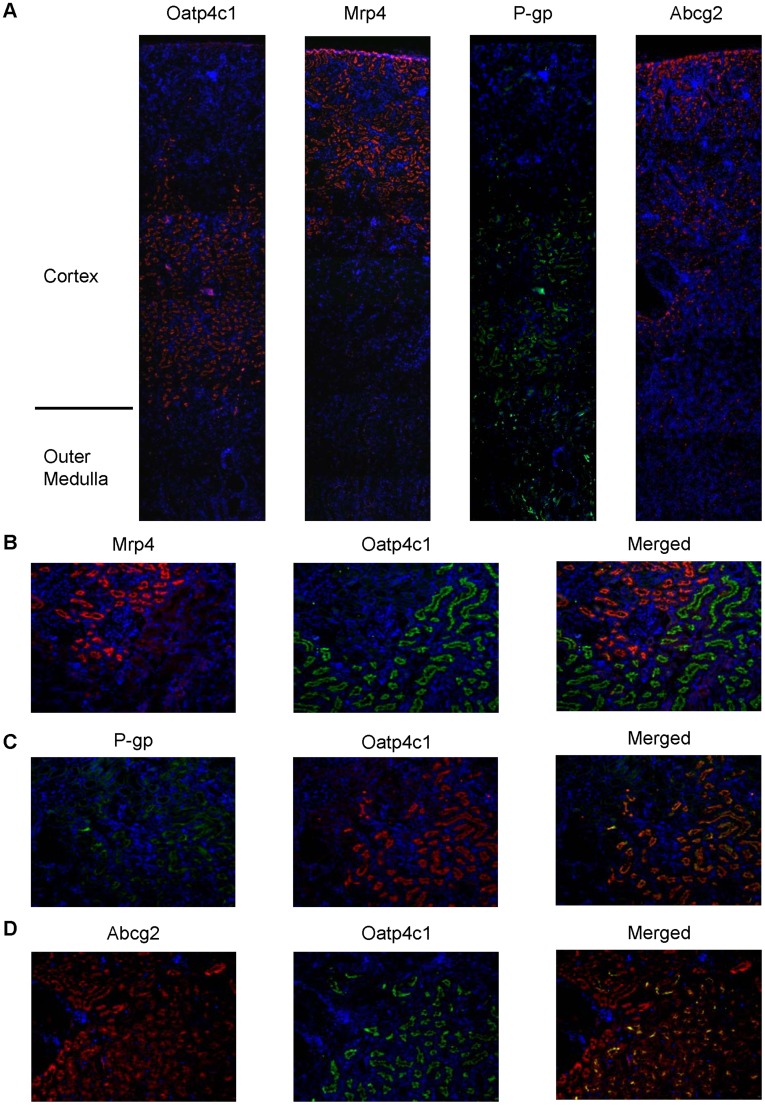
Oatp4c1 colocalization with major efflux transporters in the kidney cortex. Paraformaldehyde-fixed paraffin-embedded rat kidney sections were stained for Oatp4c1 (red), Mrp4 (red), P-gp (green), and Abcg2 (red) (A). Sections were double stained for Mrp4 (red) and Oatp4c1 (green); (C) P-gp (green) and Oatp4c1 (red); and (D) Abcg2 (red) and Oatp4c1 (green) (B). Yellow/orange color in merged panels indicates colocalization. Nuclei (blue) were stained with DAPI. Images presented in panel 5A were stitched from six individual images using NIH Image J with Fiji plugin (Magnification: 4× (A), 10× (B–D)).

### Expression of Oatp4c1 in Isolated Brush-border Membrane (BBM) and Basolateral Membrane (BLM)

Concurrently with the immunohistochemistry studies outlined in Figs. 2 and S3, we sought to definitively demonstrate the specificity of the PA1343 antibody used for western blot and immunolocalization of Oatp4c1. To this end, we isolated brush border membranes from rat kidney cortex and performed proteomic analysis on proteins collected from apical and basolateral microtubule membranes. BBM and BLM were isolated from rat kidney cortex by Percoll density gradient. Western blotting (Fig. 3A) and proteomic analysis (Fig. 3B) were applied to analyze the proteins in the two fractions. Abcg2 (∼70 KD) and Mrp4 (∼150 KD) were used as apical markers, and they were enriched in BBM. Na^+^/K^+^-ATPase (∼94 KD), a basolateral marker, was detectable in both membrane fractions, but more so in BLM than BBM. Oatp4c1 was only detected in BBM (Fig. 3A), which was consistent with the immunohistochemistry results (Fig. 2 and S3).

To validate the specificity of the PA1343 antibody, the proteome in BBM and BLM were analyzed by LC-MS/MS. The proteins in BBM and BLM were separated by SDS-PAGE. The gel bands of proteins eluting at 65–100 and 100–150 kDa were excised, and underwent reduction, alkylation, and in-gel trypsin digestion [18], [19]. The abundance of the identified proteins was determined by the total number of spectra identified from a protein (spectral counts) [23], [24], and spectral counts >2 was used as the cutoff. Spectra from proteins with molecular weight less than 65 kDa or more than 150 kDa were excluded. The LC-MS/MS analysis identified 6 proteins unique to the BBM, 19 proteins unique to the BLM, and 56 proteins were either enriched in one membrane or equally distributed in both membrane preparations. Among them, the spectral counts of the SLC and ABC family with reported localization, along are shown in Fig. 3B. The presence of the apical marker (meprin) and basolateral marker (Na^+^/K^+^-ATPase) in each membrane preparation are also shown. Eleven apical proteins were predominantly in the BBM, four basolateral proteins were enriched in the BLM, and three proteins with previously reported apical localization were found in both fractions (ambiguous). Four unique Oatp4c1 peptides (GVENPAFVPSSPDTPRRR; RASASPSQVEVSAVASR; TSQTHQNNSTSFQHMDENFGK; KVDITSTAXSPDFEAR) were found only in the BBM. These qualitative data provide additional, but conclusive evidence that Oatp4c1 localizes at the apical membrane in the rat kidney cortex.

**Figure 6:**
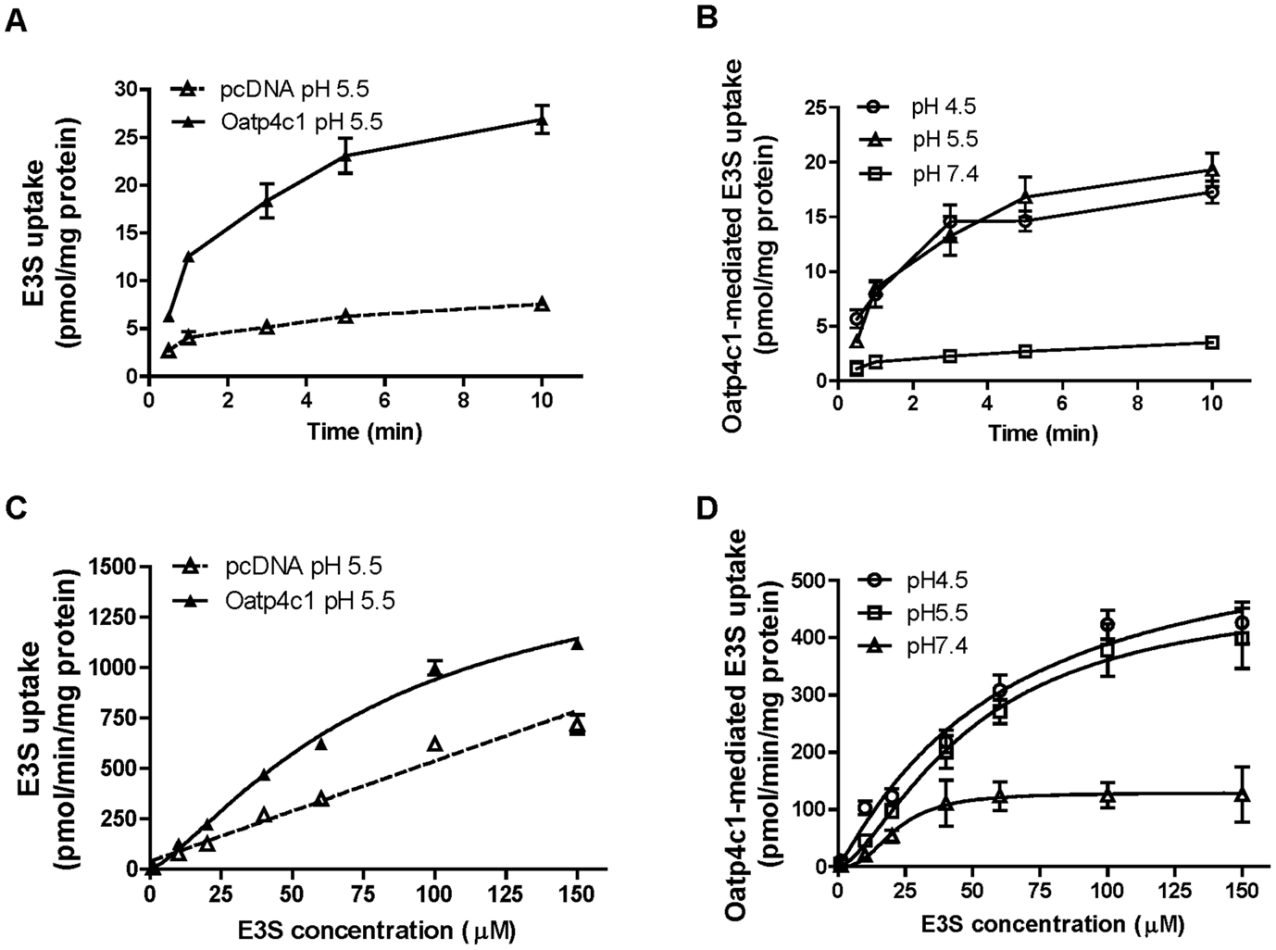
Characteristics of Oatp4c1-mediated E3S transport. Time dependent [^3^H]-E3S uptake at pH 5.5 by MDCKII-pcDNA and MDCKII-Oatp4c1 cells during incubation with 0.5 µM [^3^H]-E3S (A). Time dependent Oatp4c1 mediated uptake of [^3^H]-E3S at pH 4.5, 5.5 and 7.4 (B). Concentration dependent [^3^H]-E3S uptake in MDCKII-pcDNA and MDCKII-Oatp4c1 cells after incubation at pH 5.5 for 1 min (C). Concentration dependent Oatp4c1 mediated uptake (1 min) of [^3^H]-E3S at pH 4.5, 5.5 and 7.4 (D). GF: GF120918, MTX: methotrexate. Each point represents the mean ± S.D. of triplicates. *p<0.05, significant differences from control.

**Table 1:** Transport kinetic parameters of Oatp4c1-mediated E3S transport at pH 4.5, 5.5 and 7.4 (mean ± S.E.).

	pH 4.5	pH 5.5	pH 7.4
T_max_ [(pmol/min )/mg protein]	612.6±109.7*	484. 0±50.4***	129.3±11.0**
S_50_ [µM]	60.5±22.7	49.3±8.8	21.5±3.6
n, Hill Coefficient	1.1±0.2	1.5±0.2	2.6±1.0

The parameters were estimated by fitting a sigmoid-E_max_ model to the data. Statistical analysis was performed by two-way ANOVA with Bonferroni post-hoc test for all pairwise comparisons. *, **, *** p<0.05 indicate significant differences between pH 4.5 and 5.5, pH 4.5 and 7.4, and pH 5.5 and 7.4, respectively.

**Figure 7:**
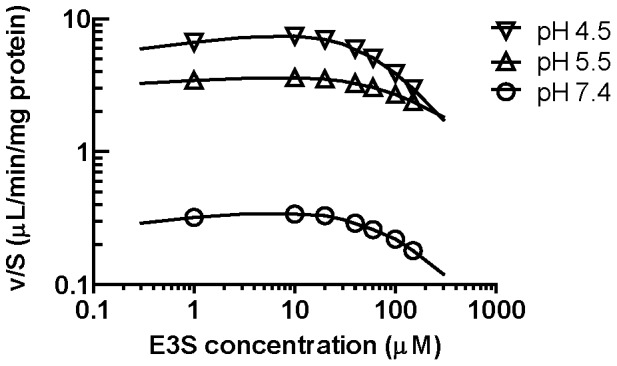
The effect of pH on uptake transport clearance. The uptake clearance 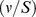 was estimated using the parameter estimates in Table 1. The solid lines show the best fit to the data using 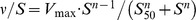 obtained from [^3^H]-E3S uptake (Fig. 6).

### Location of Oatp4c1 in the Rat Kidney

To further gain insights on the potential physiological role of Oatp4c1 we assessed its precise expression pattern at the organ and nephron level. Immunohistochemical analysis showed that Oatp4c1 is expressed densely in the renal tubules located in the juxtamedullary cortex (Fig. 4A). To ascertain its location within the tubule, we used double immunofluorescence to stain for Oatp4c1 and the proximal tubule marker, aquaporin 1 [25], or a distal tubule marker, calbindin-D_28K_ [26]. As shown in Fig. 4B and 4C, Oatp4c1 colocalizes with aquaporin 1, but not with calbindin-D_28K_. Therefore, Oatp4c1 is expressed in the proximal tubule within the juxtamedullary cortex, which suggests that it localizes primarily in the proximal straight tubules (S3). Double immunofluorescence staining was also used to access the location of Oatp4c1 relative to major apical efflux transporters in the rat kidney. As shown in Fig. 5, Mrp4 is expressed in the superficial cortex (S1, S2), P-gp is expressed in the juxtamedullary cortex (S3) and colocalizes with Oatp4c1, while Abcg2 is expressed in the whole cortex (S1, S2, S3) and partially colocalizes with Oatp4c1.

### Pharmacological Analyses of Rat Oatp4c1

To validate the functional activity of rat Oatp4c1 in MDCKII cells and to gain further insight on the role of Oatp4c1 in the transport of physiological substrates, we performed uptake and inhibition studies using E3S, which was previously shown to be a human OATP4C1 substrate [8], [9]. Leuthold et al. demonstrated that E3S uptake mediated by OATP4C1 is pH- dependent [9], but this was in contrast to the report by Mikkaichi et al. who did not observe a pH dependence in transport [5]. In preliminary studies, the uptake of [^3^H]-E3S in MDCKII-Oapt4c1 cells was susceptible to inhibition by 100 µM unlabeled E3S and transport appeared to be more efficient in cells exposed to media with pH 5.5 (Fig. S4). These data demonstrated the functionality of our *in vitro* model and provided evidence that E3S is also a substrate of rat Oatp4c1.

The transport kinetics of [^3^H]-E3S in MDCKII-Oatp4c1 were studied further to determine the pH dependent differences in transport. From a physiological perspective, relating to the expression of Oatp4c1 in the kidney, this could be important as the pH in urine ranges in the acidic range, under normal conditions, but can be basic (>pH 7), under pathological situations. Our studies showed that the time dependent uptake of [^3^H]-E3S (0.5 µM) was more efficient in Oatp4c1 cells as compared to vector transfected cells at pH 5.5 (Fig. 6A). Similar differences were obtained for pH 4.5 and pH 7.4 (data not shown). Comparatively, the net Oatp4c1 mediated uptake, obtained by subtracting the transport in vector transfected cells, was more efficient at pH 4.5 and pH 5.5 than at pH 7.4 (Fig. 6B).

**Figure 8:**
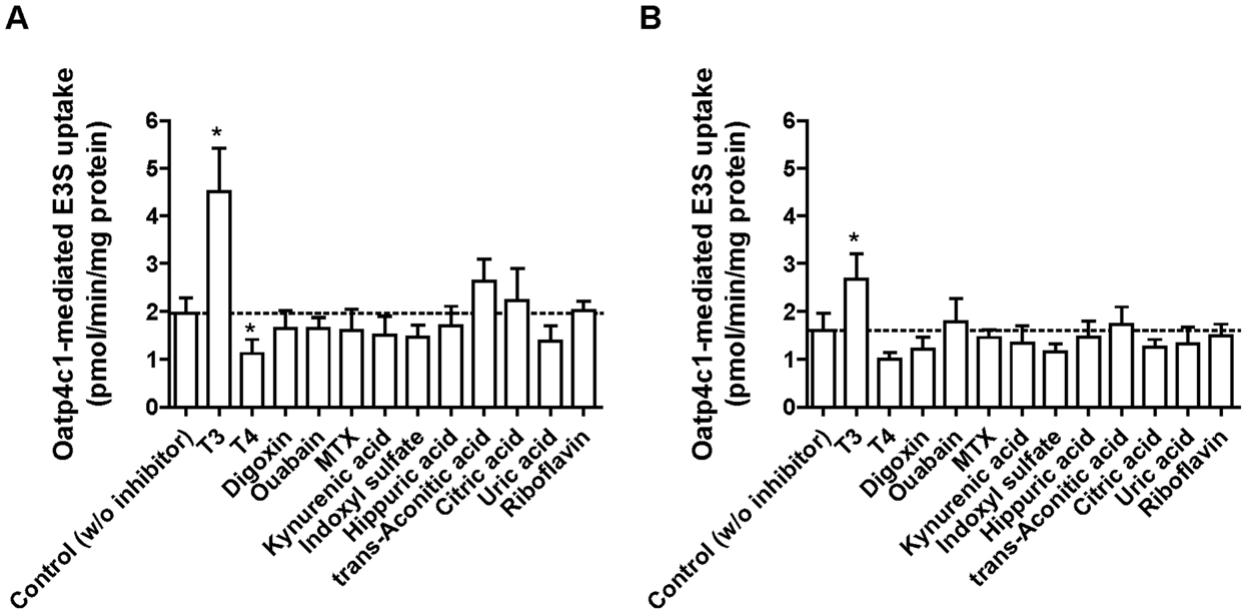
The effect of uremic toxins and anionic physiological substances on Oatp4c1-mediated E3S transport. Inhibition of Oatp4c1-mediated [^3^H]-E3S uptake (1 min) was determined in the absence (control) and presence of various compounds (100 µM) at pH 5.5 (A) and 7.4 (B). Oatp4c1-mediated uptake was estimated after subtracting the nonspecific uptake measured in pcDNA cells treated under identical conditions. In all studies the [^3^H]-E3S concentration was 0.5 µM. GF: GF120918, MTX: methotrexate. Each point represents the mean ± S.D. of triplicates. *p<0.05, significant differences from control.

To obtain quantitative estimates of the pH dependent differences in transport, we then examined the concentration dependent uptake (1 min) of [^3^H]-E3S and estimated transport kinetic parameters. Based on the time dependent uptake studies (Fig. 6B), the 1 min time point was chosen to ensure that the transport parameters were estimated during the linear phase of uptake transport. To evaluate [^3^H]-E3S transport kinetics, the initial rate of [^3^H] E3S uptake were determined for concentrations ranging from 1 to 150 µM. The [^3^H]-E3S uptake in MDCKII-Oatp4c1 and MDCKII-pcDNA cells at pH 5.5 is shown in Fig. 6C, and the net uptake at pH 4.5, 5.5 and 7.4 is shown in Fig. 6D. A sigmoid-E_max_ model with a Hill coefficient parameter was fitted to the net Oatp4c1 transport data. Parameters (Table 1) were estimated by nonlinear regression. The Michaelis-Menten K_m_-equivalent parameter, S_50_, at pH 7.4 (21.5±3.6 µM) was comparable to the reported K_m_ (26.6±4.9 µM) for human OATP4C1 at pH 7.4 [8]. In our study, there was a trend toward increasing S_50_ with a decrease in pH while the estimated T_max_ was 5- and 4-fold higher at pH 4.5 and pH 5.5, respectively, than the T_max_ estimate at pH 7.4. In addition, the estimated Hill coefficient value of 2.6 at pH 7.4 suggests positive transport cooperativity.

Using the estimated transport parameters we then determined the uptake transport clearance as a function of [^3^H]-E3S concentration. As shown in Fig. 7 the uptake transport clearance 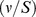 is constant at concentrations below the S_50_ value, but decreases rapidly as the substrate concentration saturates the transporter. This analysis also demonstrates that the transporter uptake clearance is 10–20 fold higher at acidic pH conditions.

Having validated E3S as an Oatp4c1 substrate, we next attempted to determine the interaction of Oatp4c1 with potential physiological substrates by studying their capacity to inhibit transport of this prototypical substrate. Mikkaichi and colleagues had previously reported that OATP4C1 transports T3 and T4 as well as ouabain and digoxin [5]. In addition, in-vivo studies by Toyohara and colleagues suggested that OATP4C1/Oatp4c1 is involved in the elimination of uremic toxins [10]. However, their report included only in vivo data without in vitro confirmation [10]. In this study we sought to confirm the interaction of these purported OATP4C1/Oatp4c1 substrates. [^3^H]-E3S (0.5 µM) uptake was determined in the absence and presence of 100 µM various compounds for 1 min at pH 5.5 (Fig. 8A) or 7.4 (Fig. 8B). [^3^H]-E3S uptake was significantly inhibited by T_4_ at pH 5.5, and significantly stimulated by the presence of T_3_ at both pH 5.5 and 7.4. In addition, [^3^H]-E3S accumulation was significantly higher with T_3_ addition than control (without T3) in both Oatp4c1-expressing and vector-transfected cells (Fig.S5). Digoxin and ouabain did not change Oatp4c1-mediated uptake. Interestingly, uremic toxins (kynurenic acid, indoxyl sulfate, hippuric acid and trans-aconitic acid), uric acid, citric acid, and riboflavin did not change Oatp4c1-mediated E3S uptake at both pH 5.5 and pH 7.4. Additional studies with either digoxin as a model substrate or by direct measurement of uremic toxin uptake in MDCKII-Oatp4c1 cells are needed to definitively determine if uremic toxins are Oatp4c1 substrates and/or inhibitors.

The driving force for Oatp4c1/OATP4C1 mediated transport is currently not known. Examination of the Oatp4c1 and OATP4C1 primary amino acid sequences by Mikkaichi and colleagues revealed that a potential ATP binding site (Walker A motif) was present [5]. However, an ATP-depletion assay revealed that OATP4C1 mediated transport of T_3_ was not ATP-dependent at pH 7.4 [5]. To examine whether rat Oatp4c1-mediated [^3^H]-E3S transport is ATP-dependent, the effect of ATP depletion on its accumulation was assessed. Cells were incubated with either uptake buffer or buffer lacking glucose, but supplemented with 20 mM 2-deoxy-D-glucose and 10 mM NaN_3_ at pH 5.5 and pH 7.4. The results showed that Oatp4c1-mediated [^3^H]-E3S uptake did not change under ATP-depleted condition (Fig. S6). In addition, [^3^H]-E3S intracellular concentrations in Oatp4c1-expressing and vector-transfected MDCKII cells were higher under ATP-depleted condition at pH 5.5 (Fig. S6A), suggesting the presence of an endogenous ATP-dependent efflux transporter, possibly Mrp1, in the MDCKII cells [27], [28].

## Discussion

In the present study, we undertook a comprehensive approach to determine the subcellular localization of Oatp4c1 in polarized epithelium. Our studies definitively demonstrated that Oatp4c1 is expressed at the apical membrane in the rat proximal tubule. Our approach included a variety of complementary methods to verify this observation. Western blotting demonstrated that the antibody used recognizes a protein of the appropriate molecular weight. Immunohistochemical and immunofluorescence analysis revealed that the protein traffics to the apical membrane of polarized epithelium in vivo and in vitro. The immunohistochemistry results were verified with four different antibodies, which were raised against unique C- and N-terminal peptides. In addition, biochemical separation of apical and basolateral membranes from polarized cells in vitro and proximal rat kidney tubules confirmed the location of Oatp4c1 at the brush border membranes by western blot and proteomic analyses. Furthermore, the proteomic analyses provide additional validation for the Oatp4c1 antibody specificity.

This extensive validation of the in vitro trafficking and in vivo subcellular localization was deemed necessary as our results were not in accordance with previous reports. Initially, Oatp4c1 was shown to localize at the basolateral membrane in the rat proximal tubule [5], and a subsequent report showed similar expression pattern of human OATP4C1 in transgenic rats harboring human SLCO4C1 [10]. These authors hypothesized that basolateral expression of Oatp4c1 worked in tandem with an apical efflux transporter to efficiently transport substrates across the kidney epithelium and into the urine [5], [10]. The contradictory finding in the original report by Mikkaichi and colleagues may have been a result of limited validation performed with the previous antibody in that study [5]. Our attempt to develop this polyclonal antibody [5], using the reported 11 amino acid peptide antigen, did not yield a reagent that was amenable to immunohistochemistry (Fig. S3). The results of the transgenic OATP4C1 expression pattern reported by Toyohara et al. [10], however, are more difficult to reconcile. In their publication, they present evidence that OATP4C1 localizes in the basolateral membrane of renal tubule epithelium in transgenic rats. Provided that the anti-OATP4C1 antibody recognized the correct transgenic protein, one potential explanation for the observed differences in the subcellular localization could be aberrant trafficking of the human OATP4C1 protein in the transgenic rats.

It is not without precedent that antibodies used to detect transporter proteins result in controversial findings [29], [30]. To avoid potential misinterpretation of results due to antibody immunoreactivity, we also applied two-dimensional LC/MS-MS analysis to identify the proteome in brush border and basolateral membranes isolated from the rat kidney cortex. The results of this study validated our western blot and immunostaining results with the polyclonal antibody PA1343. Some of the most abundant proteins, such as Na^+^/K^+^-ATPase or, rBat, SGLT2, TSC or NKCC2, were detectable in both membrane fractions, showing lack of absolute efficiency in the separation of the two membrane fractions. Cross-contamination between the two fractions was also observed in previous reports using the same method [31], [32]. However, these proteins were sufficiently enriched in the appropriate fraction suggesting adequate separation of basolateral and apical membranes. In all, the LC-MS/MS analyses identified eighteen membrane transporter proteins (Fig. 3B). Based on previous knowledge, fifteen of these proteins, including Oatp4c1, were enriched or uniquely identified in the appropriate fraction (apical or basolateral) and three proteins (ambiguous) were equally present in both preparations (Fig. 3B). Four unique Oatp4c1 sequence peptides were found only in BBM but not in BLM. These results are in agreement with the findings from western blotting and immunohistochemical analyses and definitively demonstrate that Oatp4c1 is localized at the apical membrane in the rat proximal tubule.

Previous studies have shown the expression of several organic anion transporters in the kidney [3], [33], [34], [35], [36], [37], [38]. These transporters include: Oatp1a1 (previously Oatp1), Oatp1a3v1 (previously OAT-K1), Oatp1a3v2 (previously OAT-K2), Oatp1a5 (previously Oatp3), and Oatp2a1 (previously PGT). Oatp1a1 is expressed at the apical membrane of the proximal tubule S3 segment in the outer medulla [3]. Oatp1a3v1 was detected in the brush-border membranes isolated from rat kidney by western blotting [4], and both of Oatp1a3v1 and Oatp1a3v2 showed a functionally apical uptake in cell systems [39]. Oatp1a5 mRNA is highly expressed in rat kidney [40], but its subcellular localization has not been determined yet. Oatp2a1 localizes at the apical surface of rat collecting ducts, glomerular endothelial and mesangial cells, but not in proximal tubules [34]. Our biochemical membrane separation and LC-MS/MS analysis of BBM and BLM isolated from rat kidney cortex, which is rich in proximal tubules, demonstrated that Oatp1a3 was only detected in BBM not in BLM (Figure 3B). This was in accord with previous reports [4]. Additionally, a single peptide of Oatp1a1 was detected in the BBM, but that observation was below the threshold limit of 2 unique peptides and was not included in Figure 3B. The functional reason for colocalization of Oatp1a3 with Oatp4c1 is not obvious and additional studies are required to determine if these proteins have common substrates and the relative affinity differences for those substrates. In contrast, OATP4C1 is the only reported OATP in human proximal tubules. The different expression profile between rat and human suggests the importance of OATP4C1 and indicates potential difficulties in interspecies scaling and extrapolation.

In this study, we found Oatp4c1 is expressed primarily at the apical membrane in the proximal straight tubule (S3), suggesting its potential role in renal reabsorption of substrates. In agreement with previous studies, we showed that E3S is an Oatp4c1 substrate. Oatp4c1-mediated E3S uptake was inhibited by T_4_ and enhanced by T_3_, suggesting that T_4_ is a substrate/inhibitor and T_3_ is an enhancer. Both T_3_ and T_4_ have been reported as OATP4C1 substrates [5], and Yamaguchi et al. has shown that T_3_ decreases OATP4C1-mediated E3S transport at pH 7.4 [8]. Although this is not consistent with our data, it may be attributed to differences between the rat Oatp4c1 and human OATP4C1, which share 80.4% homology. However, digoxin, ouabain, and MTX, which are reported OATP4C1 substrates, did not change Oatp4c1-mediated E3S uptake in our current work. This is consistent with the results of Yamaguchi et al. in MDCKII-OATP4C1 cells who showed that E3S uptake was not inhibited by digoxin and vice versa [8]. A similar observation was made for T_3_ and digoxin [5], [8]. Collectively, these data provide evidence that OATP4C1, and possibly Oatp4c1, possess multiple substrate recognition sites. One recognizes E3S/T_3_, and another that recognizes digoxin. The identification of Oatp4c1 substrates should be investigated further so that its physiological role as well as its function in the renal disposition of drugs and/or their metabolites can be understood.

With respect to physiological substrates, Toyohara and colleagues showed that OATP4C1 transgenic rats had decreased uremic toxin plasma concentrations in the 5/6 nephrectomy renal failure model as compared to the same model established wild-type rats [10]. The authors suggested that this is consistent with the model of basolateral uptake by OATP4C1 and apical efflux by another transporter. However, in this study, we demonstrated that Oatp4c1 is expressed in the apical membranes and functions as an uptake transporter, thereby suggesting a function in renal reabsorption, not tubular secretion. Therefore, there may be other basolateral uptake or apical efflux transporters involved in renal elimination of uremic toxins. For example, Oat1 and Oat3, which are expressed at basolateral membrane in proximal tubule [41], have been shown to be responsible for indoxyl sulfate and hippuric acid uptake from blood into tubular cells [42]. Moreover, the down-regulation of Oat1 and Oat3 expression in chronic renal failure model may result in the uremic toxin accumulation [43]. As for the role of apical efflux transporters, hippuric acid, indoxyl sulfate and kynurenic acid have been shown to inhibit MRP4-mediated MTX uptake and ABCG2-mediated E3S uptake in inverted membrane vesicles [44]. Furthermore, ABCG2-mediated transport is less efficient at higher pH (i.e., >pH 7.0) [45], [46], which may prevail under pathophysiological conditions in the proximal tubules. Thus, impaired efflux by an ABC transporter may also provide an explanation for increased uremic toxin accumulation in the blood of animals in the renal failure models [10]. Clearly, more studies are required to elucidate the role of different transporters in human kidney pathophysiology and in the animal models of renal failure.

The pH dependence of OATP mediated transport has been demonstrated in several rodent and human OATP proteins [9], [47], [48], [49]. However, the driving force responsible for Oatp4c1 mediated transport is not fully understood. Mikkaichi et al. showed that pH did not affect OATP4C1-mediated uptake [5], while Leuthold et al. showed that E3S and T_3_ uptake are significantly higher at extracellular pH 6.5 than pH 8.0 in *Xenopus laevis* oocytes expressing OATP4C1 [9]. In this study, we demonstrated that Oatp4c1-mediated E3S uptake was pH dependent. In our estimates, T_max_ was 4-fold higher at pH 5.5 than at pH 7.4 and uptake clearance was 10–20 fold higher at acidic pH. The observed pH-dependent Oatp4c1-mediated uptake may be a consequence of altered ionization state of the substrate molecule. However, with a pKa of 2 [45], the ionization state of E3S is essentially constant under our experimental conditions (pH 4.5–7.4). Therefore, any changes in E3S uptake from pH 4.5 to 7.4 should be a result of the pH gradient and may involve HCO_3_^−^ exchange. Further studies examining substrate uptake in HCO_3_^−^ replete and depleted conditions are needed to confirm the potential role of an organic anion/HCO_3_^−^ exchange mechanism. In addition, the pH in renal proximal tubules of rats is acidic [50], [51], [52], [53] and pH dependent changes in protein structure may also be important for substrate transport [45].

In conclusion, our data collectively demonstrate that rat Oatp4c1 polarizes to the apical membranes in *in vitro* cell models (transfected MDCKII, LLC-PK1 cells), as well as in the rat kidney proximal tubules, which suggests that Oatp4c1 is involved in renal reabsorption. [9]The Oatp4c1-mediated E3S uptake is ATP-independent and pH-dependent, suggesting that pH gradient could be the driving force for Oatp4c1 mediated transport. In addition, our transport data corroborate previous data that Oatp4c1 possesses multiple binding sites, but these results require further verification.

## Supporting Information

Figure S1 **Immunolocalization of Oatp4c1 in polarized LLC-PK1 cells.** Cells were double stained with Oatp4c1 (red) and ZO-1 (green). Nuclei were stained with DAPI (blue). Center image in the Oatp4c1 panel is a single optical section of the x–y plane while top and right images represent x–z and y–z planes, respectively, reconstructed from image stacks. The apical and basal sides can be demarcated by ZO-1 and the nuclei, respectively, in both x–z and y–z sections.
(TIFF)

Figure S2 **Effect of sodium butyrate (NaB) on Oatp4c1 expression and subcellular localization in MDCKII cells.** MDCKII-pcDNA and MDCKII-Oatp4c1 cells were treated with 1 or 5 mM NaB for 24 hr. Oatp4c1 expression (red) was assessed in cells grown in monolayers (A) and cell pellets were collected, lysed and subjected to PCR for Slco4c1 mRNA expression (B) or western blot for Oatp4c1 protein expression (C). 18 s and β-actin were used as loading controls in panels B and C, respectively. Oatp4c1 subcellular localization was assessed in polarized MDCKII cells by confocal microscopy (D). After treatment with 5 mM NaB for 24 hr, cells were double stained with Oatp4c1 (red) and ZO-1 (green). Nuclei were stained with DAPI (blue). Center image in the Oatp4c1 panel is a single optical section of the x–y plane while top and right images represent x–z and y–z planes, respectively, reconstructed from image stacks. The apical and basal sides can be demarcated by ZO-1 and the nuclei, respectively, in both x–z and y–z sections.
(TIFF)

Figure S3 **Apical Oatp4c1 localization in rat kidney tubules was verified by four different antibodies.** Paraformaldehyde-fixed paraffin-embedded rat kidney tissue sections were stained with different rabbit polyclonal anti-Oatp4c1 antibodies, as indicated. Color development with NovaRed signifies Oatp4c1 staining. All sections were counterstained with hematoxylin. Rabbit IgG was used as a negative control. Antibody specificity (PA1343) was also demonstrated by pre-absorbing the antibody with antigen peptide (STITVEEDLNKIENEG) overnight at 4°C prior to use. PA1556 was generated against the peptide (SPDFEARAGKC) previously reported by Mikkaichi and colleagues [5].
(TIFF)

Figure S4 **Oatp4c1 mediated uptake of [^3^H]-E3S is inhibited by E3S.** MDCKII-pcDNA and MDCKII-Oatp4c1 cells were incubated with 0.5 µM [3 H]-E3S in the absence (control) and presence of 100 µM unlabeled E3S for 1 min at pH 5.5 (black bars) and 7.4 (white bars). Oatp4c1 mediated uptake was calculated after subtraction of nonspecific uptake by pcDNA cells. Each column represents the mean ± S.D. of triplicates. Statistical analysis was performed with unpaired student’s t-test. *p<0.05, significant differences from control.
(TIFF)

Figure S5 **Inhibition of [^3^H]-E3S uptake by various compounds.** MDCKII-pcDNA and MDCKII-Oatp4c1 cells were incubated with 0.5 µM [^3^H]-E3S in the absence (control) and presence of various compounds (100 µM) for 1 min at pH 5.5 (A) and 7.4 (B). Each point represents the mean ± S.D. of triplicates.
(TIFF)

Figure S6 **Effect of ATP on [^3^H]-E3S uptake via Oatp4c1.** (A) MDCKII-pcDNA (white bars) and MDCKII-Oatp4c1 cells (black bars) were incubated with 0.5 µM [^3^H]-E3S for 1 min at pH 5.5 and pH 7.4. Twenty minutes prior to the transport experiment, and for the duration of transport, cell medium was replaced with medium that contained 20 mM 2-deoxy-D-glucose and 10 mM NaN_3_ without D-glucose. (B) Oatp4c1-mediated uptake was calculated after subtraction of nonspecific uptake by pcDNA cells. Each column represents the mean ± S.D. of triplicates.
(TIFF)
