# Supplementary figures and images for: Localization and Functional Characterization of the Rat Oatp4c1 Transporter in an In Vitro Cell System and Rat Tissues
Source: PLoS One. 2012 Jun 29;7(6):e39641. doi: 10.1371/journal.pone.0039641 (PMC3387246; doi:10.1371/journal.pone.0039641)

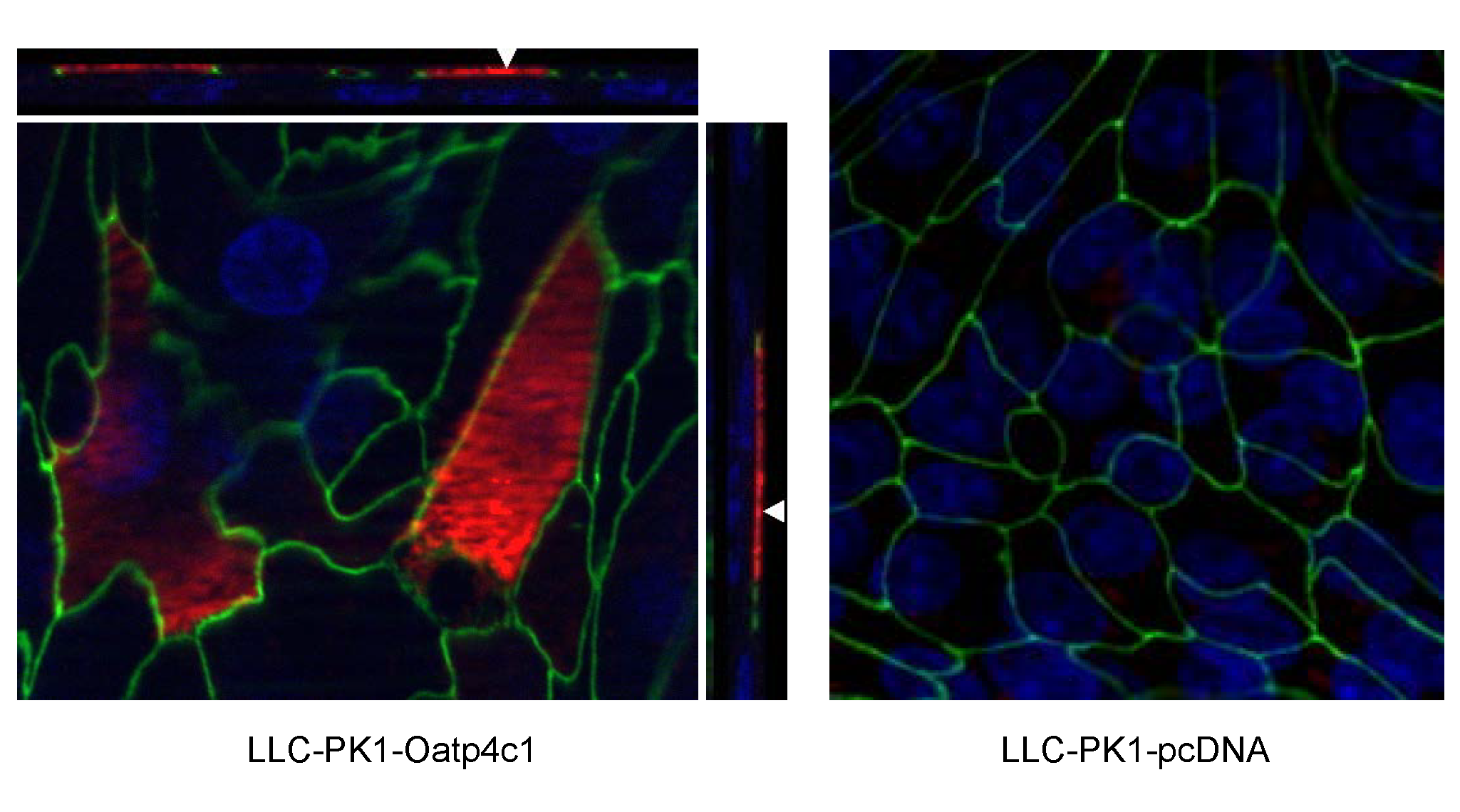

Supplement: Figure S1 — Immunolocalization of Oatp4c1 in polarized LLC-PK1 cells. Cells were double stained with Oatp4c1 (red) and ZO-1 (green). Nuclei were stained with DAPI (blue). Center image in the Oatp4c1 panel is a single optical section of the x–y plane while top and right images represent x–z and y–z planes, respectively, reconstructed from image stacks. The apical and basal sides can be demarcated by ZO-1 and the nuclei, respectively, in both x–z and y–z sections. (TIFF) [file pone.0039641.s001.tif]

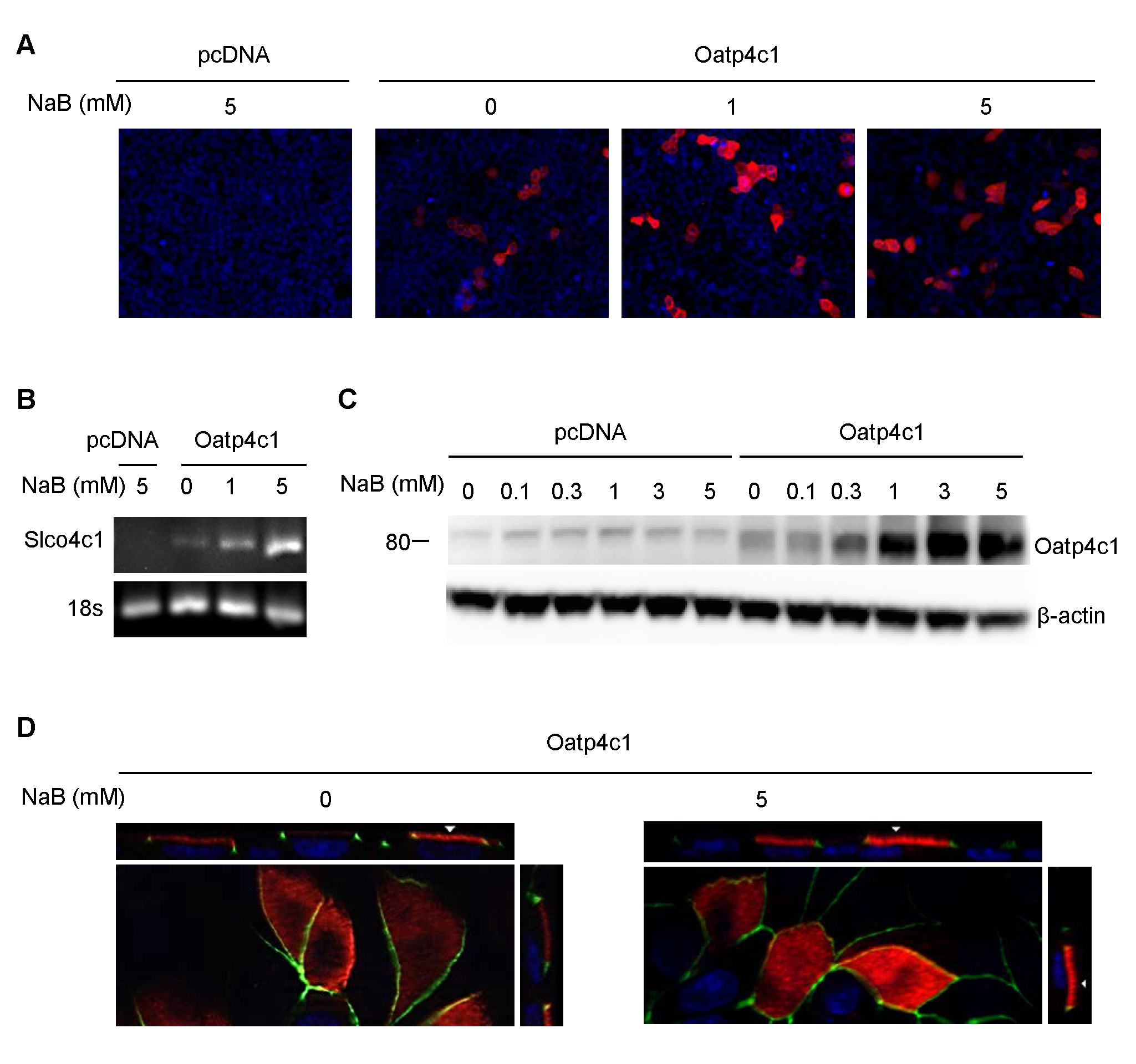

Supplement: Figure S2 — Effect of sodium butyrate (NaB) on Oatp4c1 expression and subcellular localization in MDCKII cells. MDCKII-pcDNA and MDCKII-Oatp4c1 cells were treated with 1 or 5 mM NaB for 24 hr. Oatp4c1 expression (red) was assessed in cells grown in monolayers (A) and cell pellets were collected, lysed and subjected to PCR for Slco4c1 mRNA expression (B) or western blot for Oatp4c1 protein expression (C). 18 s and β-actin were used as loading controls in panels B and C, respectively. Oatp4c1 subcellular localization was assessed in polarized MDCKII cells by confocal microscopy (D). After treatment with 5 mM NaB for 24 hr, cells were double stained with Oatp4c1 (red) and ZO-1 (green). Nuclei were stained with DAPI (blue). Center image in the Oatp4c1 panel is a single optical section of the x–y plane while top and right images represent x–z and y–z planes, respectively, reconstructed from image stacks. The apical and basal sides can be demarcated by ZO-1 and the nuclei, respectively, in both x–z and y–z sections. (TIFF) [file pone.0039641.s002.tif]

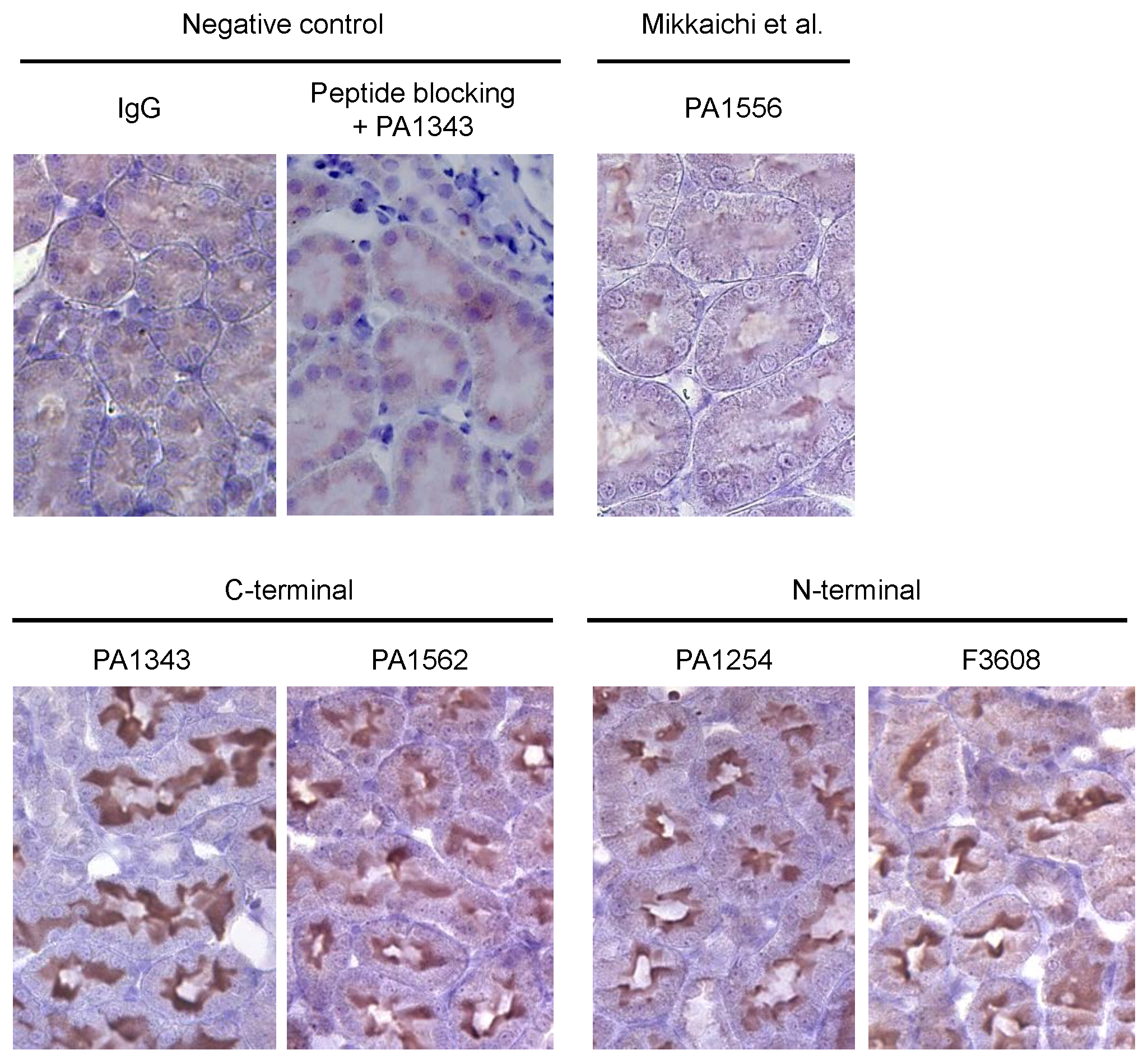

Supplement: Figure S3 — Apical Oatp4c1 localization in rat kidney tubules was verified by four different antibodies. Paraformaldehyde-fixed paraffin-embedded rat kidney tissue sections were stained with different rabbit polyclonal anti-Oatp4c1 antibodies, as indicated. Color development with NovaRed signifies Oatp4c1 staining. All sections were counterstained with hematoxylin. Rabbit IgG was used as a negative control. Antibody specificity (PA1343) was also demonstrated by pre-absorbing the antibody with antigen peptide (STITVEEDLNKIENEG) overnight at 4°C prior to use. PA1556 was generated against the peptide (SPDFEARAGKC) previously reported by Mikkaichi and colleagues [5]. (TIFF) [file pone.0039641.s003.tif]

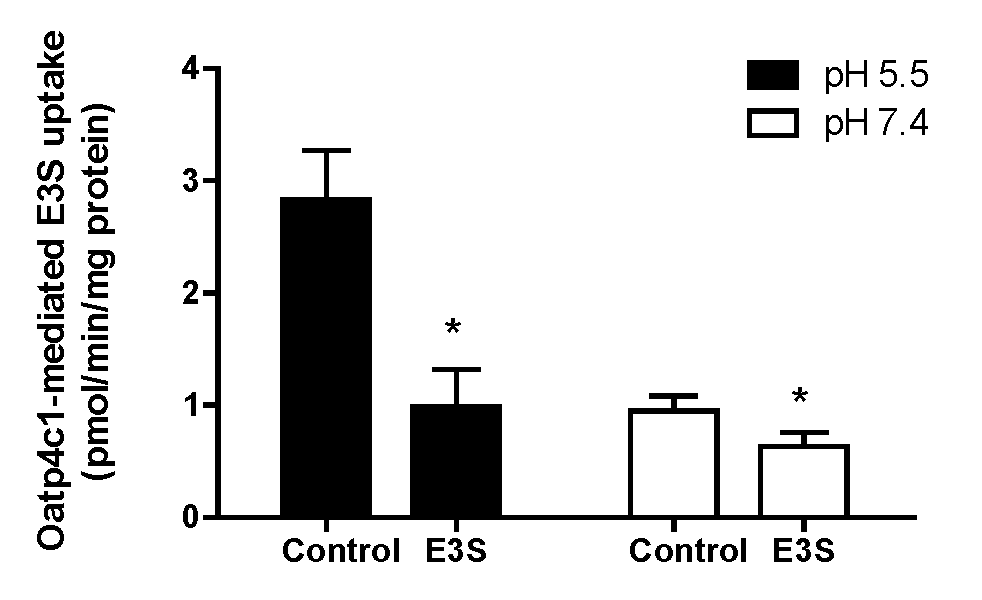

Supplement: Figure S4 — Oatp4c1 mediated uptake of [3H]-E3S is inhibited by E3S. MDCKII-pcDNA and MDCKII-Oatp4c1 cells were incubated with 0.5 µM [3 H]-E3S in the absence (control) and presence of 100 µM unlabeled E3S for 1 min at pH 5.5 (black bars) and 7.4 (white bars). Oatp4c1 mediated uptake was calculated after subtraction of nonspecific uptake by pcDNA cells. Each column represents the mean ± S.D. of triplicates. Statistical analysis was performed with unpaired student’s t-test. *p<0.05, significant differences from control. (TIFF) [file pone.0039641.s004.tif]

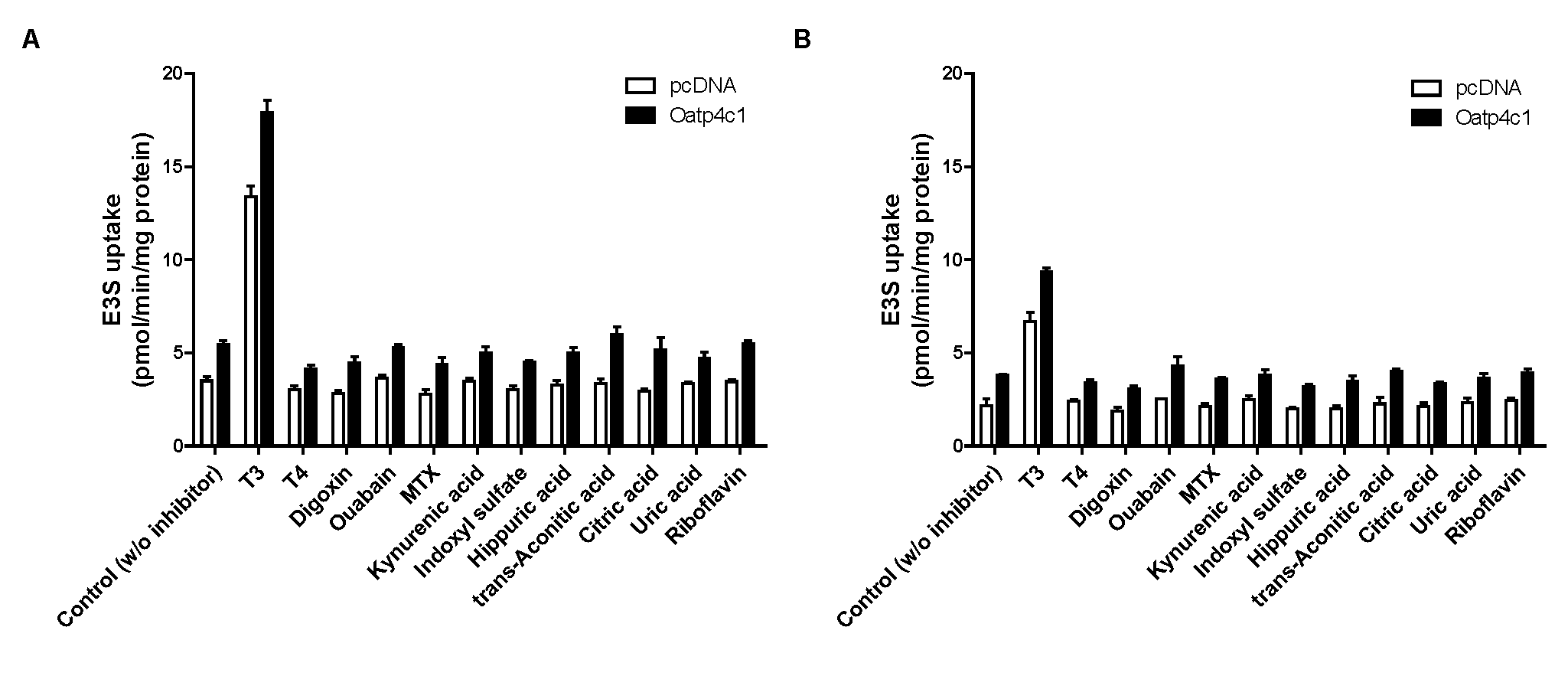

Supplement: Figure S5 — Inhibition of [3H]-E3S uptake by various compounds. MDCKII-pcDNA and MDCKII-Oatp4c1 cells were incubated with 0.5 µM [3H]-E3S in the absence (control) and presence of various compounds (100 µM) for 1 min at pH 5.5 (A) and 7.4 (B). Each point represents the mean ± S.D. of triplicates. (TIFF) [file pone.0039641.s005.tif]

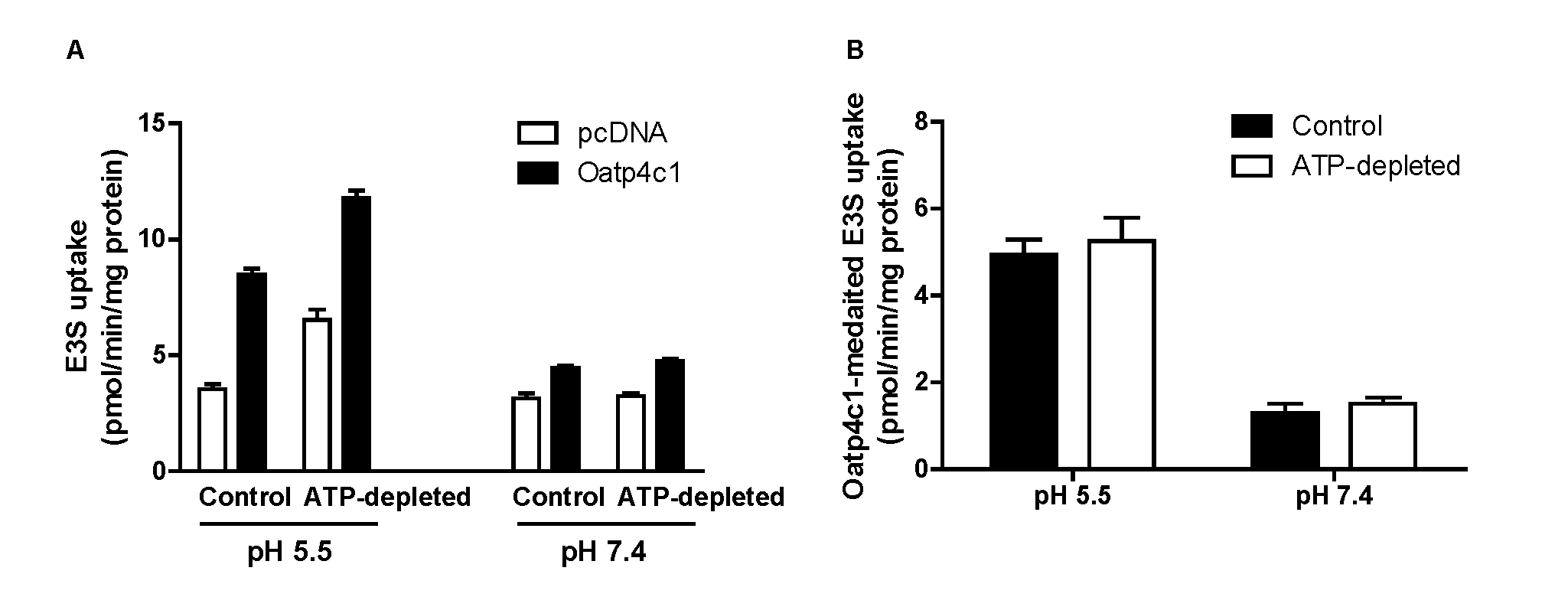

Supplement: Figure S6 — Effect of ATP on [3H]-E3S uptake via Oatp4c1. (A) MDCKII-pcDNA (white bars) and MDCKII-Oatp4c1 cells (black bars) were incubated with 0.5 µM [3H]-E3S for 1 min at pH 5.5 and pH 7.4. Twenty minutes prior to the transport experiment, and for the duration of transport, cell medium was replaced with medium that contained 20 mM 2-deoxy-D-glucose and 10 mM NaN3 without D-glucose. (B) Oatp4c1-mediated uptake was calculated after subtraction of nonspecific uptake by pcDNA cells. Each column represents the mean ± S.D. of triplicates. (TIFF) [file pone.0039641.s006.tif]
